# Dihydrocelastrol induces antitumor activity and enhances the sensitivity of bortezomib in resistant multiple myeloma by inhibiting STAT3-dependent PSMB5 regulation: Anti-myeloma activity of a novel dihydro-analog compound in bortezomib-resistant cells

**DOI:** 10.3724/abbs.2023260

**Published:** 2023-11-27

**Authors:** Shuhan Jin, Bo Li, Bibo Zhang, Xuejie Gao, Xinyan Jia, Li Xu, Shuaikang Chang, Ke Hu, Guanli Wang, Zhijian Xu, Ting Zhang, Dongliang Song, Guang Yang, Xiaosong Wu, Huabin Zhu, Cheng Huang, Yumeng Lu, Jumei Shi, Weiliang Zhu, Gege Chen

**Affiliations:** 1 Department of Hematology Shanghai East Hospital Tongji University School of Medicine Shanghai 200120 China; 2 State Key Laboratory of Drug Research Drug Discovery and Design Center Shanghai Institute of Materia Medica Chinese Academy of Sciences Shanghai 201203 China; 3 Department of Hematology Shanghai Tenth People’s Hospital Tongji University School of Medicine Shanghai 200072 China; 4Department of Hematology the Affiliated People’s Hospital of Ningbo University Ningbo 315000 China

**Keywords:** dihydrocelastrol, multiple myeloma, JAK2/STAT3, PSMB5, bortezomib resistance

## Abstract

Multiple myeloma (MM) is characterized by excessive aggregation of B-cell-derived malignant plasma cells in the hematopoietic system of bone marrow. Previously, we synthesized an innovative molecule named dihydrocelastrol (DHCE) from celastrol, a triterpene purified from medicinal plant *Tripterygium wilfordii*. Herein, we explore the therapeutic properties and latent signal transduction mechanism of DHCE action in bortezomib (BTZ)-resistant (BTZ-R) MM cells. In this study, we first report that DHCE shows antitumor activities *in vitro* and *in vivo* and exerts stronger inhibitory effects than celastrol on BTZ-R cells. We find that DHCE inhibits BTZ-R cell viability by promoting apoptosis via extrinsic and intrinsic pathways and suppresses BTZ-R MM cell proliferation by inducing G0/G1 phase cell cycle arrest. In addition, inactivation of JAK2/STAT3 and PI3K/Akt pathways are involved in the DHCE-mediated antitumor effect. Simultaneously, DHCE acts synergistically with BTZ on BTZ-R cells. PSMB5, a molecular target of BTZ, is overexpressed in BTZ-R MM cells compared with BTZ-S MM cells and is demonstrated to be a target of STAT3. Moreover, DHCE downregulates PSMB5 overexpression in BTZ-R MM cells, which illustrates that DHCE overcomes BTZ resistance through increasing the sensitivity of BTZ in resistant MM via inhibiting STAT3-dependent PSMB5 regulation. Overall, our findings imply that DHCE may become a potential therapeutic option that warrants clinical evaluation for BTZ-R MM.

## Introduction

Multiple myeloma (MM) is distinguished by clonal aggregation of malignant plasma cells in the hematopoietic system [1]. In 2003, bortezomib (BTZ), as the first proteasome inhibitor, was licensed by the US Food and Drug Administration for the therapy of multiple myeloma [2]. BTZ is an antitumor drug employed for the management of patients with MM and mantle cell lymphoma, either alone or in combination with lenalidomide, dexamethasone, and melphalan [ 3, 4]. BTZ produces a marked effect through inhibiting the 26S proteasome, which regulates intracellular protein degradation [5]. The 26S proteasome has a 20S cylindrical core particle (CP) and 19S regulatory particles (RPs). CP, in a configuration similar to a hollow cylinder, is made up of four stacked rings [6]. The interior rings of the two identical subunits carried by the catalytic residues of active sites are encoded by the proteasome subunit beta (PSMB) genes: the proteasome subunit β5 (chymotrypsin-like, PSMB5), the proteasome subunit β2 (trypsin-like, PSMB2), and the proteasome subunit β1 (caspase-like, PSMB1). Notably, BTZ is targeted to reversibly suppress the chymotrypsin-like activity of the PSMB5 subunit. This mechanism allows BTZ to decrease excessive protein degradation in MM, leading to growth inhibition and apoptosis [7]. However, resistance and/or intolerance to BTZ is a non-ignorable clinical challenge in the treatment of MM. According to research, relapsed and/or refractory MM patients who had previously undergone BTZ therapy had a lower overall response rate than BTZ-­naive patients [8]. Hence, new agents and strategies to enhance the clinical prognosis are critically warranted.

Celastrol is one of the medicinal ingredients extracted from “Thunder of God Vine”, a Chinese medicine ( *Tripterygium wilfordii* Hook F.). This natural substance, which has been proven to be antioxidant or anti-inflammatory, has been utilized to treat asthmatics, chronic inflammation, neurological disease, and systemic lupus erythematosus [9]. Furthermore, previous research has shown that celastrol suppresses cancer cell growth and induces leukemic cell death in various malignancies, such as breast cancer [10], leukemia [11], osteosarcoma [12], prostate cancer [9], and MM [13].

Dihydrocelastrol (DHCE **)** is an entirely new compound produced by our group based on the structure of celastrol. Previously, we confirmed that DHCE exerts potent antitumor activity in BTZ-sensitive (BTZ-S) MM [14] and mantle cell lymphoma cells [15]. Here, we further investigated the anti-myeloma effectiveness and associated pathway of DHCE action against BTZ-resistant (BTZ-R) MM *in vitro* and *in vivo*.

## Materials and Methods

### Cells and cell culture

The human BTZ-S MM cell lines NCI-H929 and RPMI-8226 were acquired from American Type Culture Collection (Manassas, USA). Over an 8-month period, we gradually increased extracellular concentrations of BTZ to produce the BTZ-R NCI-H929R and RPMI-8226R5 cell lines. The MM cell lines were maintained in RPMI-1640 medium with 10% fetal bovine serum (FBS; GIBCO, Carlsbad, USA), 1% streptomycin, and 1% penicillin, refreshed every two days. The culture environment was maintained at 37°C in 5% CO _2_. Blood samples from three healthy normal human volunteers and peripheral blood mononuclear cells (PBMCs) were isolated by Ficoll-Hypaque density gradient centrifugation at 800 *g* for 25 min at room temperature. PBMCs (4×10 ^5^ cells/mL) were plated in RPMI-1640 with 20% FBS, 1% streptomycin, and 1% penicillin. This study was approved by the Institutional Ethics Committee of Shanghai Tenth People’s Hospital. Each healthy volunteer provided informed consent.

### Reagents and antibodies

DHCE was synthesized as previously described [14] and dissolved in dimethyl sulfoxide (DMSO; Sigma, St Louis, USA) to make a stock solution of 4 mM. The stock solution was kept at –20°C. In the following experiments, DHCE was diluted to the needed concentrations in the cell suspension. Cell Counting Kit-8 (CCK-8) was purchased from Shanghai Yeasen Biotechnology Co., Ltd. (Shanghai, China). The pan-caspase inhibitor Z-VAD-FMK was obtained from Selleck Chemicals (Houston, USA). The eBioscience™ Annexin V-FITC Apoptosis Detection Kit was acquired from Thermo Fisher Scientific (Carlsbad, USA). The Cell-Light EdU Apollo 488 *In Vitro* Imaging Kit was obtained from RiboBio (Guangzhou, China). PI/RNase Staining Buffer was purchased from BD Pharmingen (Franklin Lakes, USA). Antibodies against CDK4, CDK6, Bcl-2, Bcl-xl, and cyclin D1 were purchased from Abcam (Cambridge, USA). Antibodies targeting cleaved caspase-8, caspase-9, JAK2, phospho-JAK2, STAT3, phospho-STAT3, PARP, phospho-PI3K, phospho-Akt, c-Myc, and PSMB5 were acquired from Cell Signaling Technology (Beverly, USA).

### Cell viability assay

NCI-H929R and RPMI-8226R5 cells (2×10 ^5^ cells/mL) were cultured in 96-well plates at 95 μL/well. Subsequently, the cell suspension was exposed to 5 μL/well of DHCE at various doses (0, 1, 2, 3, 4, or 5 μM) for 24, 48, and 72 h. Additionally, BTZ-R MM cells were treated with DHCE, BTZ, or a combination of both in a constant ratio. After incubation at 37°C in 5% CO _2_, 10 μL CCK-8 was added to each sample. After incubation for an additional 2 h at 37°C in 5% CO _2_, the absorbances of all samples were recorded at 450 nm with a 96-well plate microplate reader. The half-maximal inhibitory concentration (IC _50_) and the combination index (CI) were calculated using CalcuSyn software (Biosoft, Ferguson, USA). The CI value <1 implies a synergetic effect of the drug combination [16] .

### Soft agar assay of colony formation

BTZ-R MM cells (2×10 ^3^ cells/well) pretreated with 0, 2, or 4 μM DHCE were mixed with 10% FBS and 0.33% agar. Then, cell suspensions were plated onto a solidifying stratum of 0.5% agar in 6-well plates. After 2 weeks of incubation at 37°C in 5% CO _2_, we observed macroscopic evidence of cell colonies. Then, the agar was stained with 1% crystal violet in the dark for 1 h. With the ImageJ software (NIH, Bethesda, USA), colonies were quantified after being photographed with a digital camera.

### 5-Ethynyl-2′-deoxyuridine (EdU) labeling and immunofluorescence microscopy

NCI-H929R and RPMI-8226R5 cells (2×10 ^5^ cells/mL) were cultured with or without 2 μM DHCE in a 6-well plate, and 10 μM EdU was added to the cell suspension. After incubation for 48 h at 37°C in 5% CO _2_, the cells were harvested prior to fixation in 4% paraformaldehyde for 20 min. Then, 2 mg/mL glycine was added to neutralize the solution. Subsequently, the cells were treated with 0.5% Triton X-100 for 10 min at room temperature (RT). With three subsequent rinses in phosphate buffered saline (PBS), cells were treated with 100 μL of 1× Apollo reaction solution for 30 min, protected against exposure to light at RT, followed by treatment with DAPI at RT for 10 min. Finally, cells were observed under a DM6000B confocal laser scanning microscope (Leica, Heidelberg, Germany).

### Cell cycle analysis

After 48 h of exposure to 0, 2, or 4 μM DHCE, BTZ-R MM cells (2×10 ^5^ cells/mL) were washed with PBS. The cells underwent fixation in cold 70% ethanol at –20°C for more than 24 h. After fixing and permeabilization, the cell samples were incubated with PI/RNase staining buffer (200 μL/test) for 10 min protected from light exposure. A flow cytometer (BD FACS Canto II; BD, San Diego, USA) was used to examine the stained samples. ModFit LT software was used to analyze the data.

### Apoptosis measurement

NCI-H929R and RPMI-8226R5 cells (2×10 ^5^ cells/mL) were cultured with DHCE (0, 1, 2, or 4 μM), Z-VAD-FMK (40 μM) or in combination. After 48 h, the cells were harvested and then rinsed with PBS. Then, 2.5 μL Annexin V-FITC and 50 μL binding buffer (1×) were introduced to each sample. After mixing and incubation for 20 min in the dark, the cells were mixed with 5 μL propidium iodide (20 μg/mL) and 250 μL binding buffer (1×). The stained samples were then observed with a flow cytometer (BD FACS Canto II). The collected results of different samples were visualized and assessed using FlowJo 10 (Tree Star, Ashland, USA).

### Western blot analysis

NCI-H929 and RPMI-8226 cells, along with NCI-H929R and RPMI-8226R5 cells, were exposed to varying DHCE concentrations (0, 2, or 4 μM). After 48 h, the cells were lysed on ice in ice-cold lysis buffer (100 mM Tris-HCl, 4% SDS, and 20% glycerol, pH 6.8). Subsequently, the supernatant was aspirated after 30 min of continuous agitation at 4°C. A BCA Protein Assay Kit (Beyotime, Shanghai, China) was employed for protein quantification of different samples. Equal amount of protein samples (30 μg/lane) were separated by 8%–15% sodium dodecyl sulfate-polyacrylamide gel electrophoresis (SDS-PAGE). The proteins were transferred to either nitrocellulose or PVDF membranes, and the membranes were blocked in 5% nonfat dry milk or 5% bovine serum albumin (BSA) overnight at 4°C. After being rinsed three times (10 min each) with PBST (1× PBS+0.01% Tween 20), the membrane was incubated with the diluted primary antibody overnight at 4°C, followed by incubation with HRP-conjugated secondary antibody for 1 h at room temperature. Protein bands were visualized using enhanced chemiluminescence (ECL) reagent and images were acquired, recorded and analyzed with a western blot detection imaging system (ImageQuant 800; Amersham, Buckinghamshire, UK).

### Tumor xenograft model

A total of 8 male nude mice (athymic nu/nu BALB/c, 6 weeks, 18–20 g) were purchased from Shanghai Laboratory Animal Center (Shanghai, China). Mice were kept in an SPF-class housing laboratory and provided with a regular meal and unrestricted access to water. NCI-H929R MM cells (4×10 ^8^ cells/mL) in serum-free culture medium were mixed at a 1:1 ratio with Matrigel (Corning, New York, USA) as directed. The nude mice were given injections subcutaneously in the upper flank area. When the tumors reached a length of approximately 5 mm, the mice were arbitrarily separated into two groups: the control group (saline, daily) and DHCE group (15 mg/kg DHCE in 5% DMSO, 15% Tween-80, and saline, daily). The tumor size and body weight were measured daily. Tumor volume was estimated as follows: V=length×width ^2^/2. When the tumors of the control group reached approximately 2000 mm ^3^ after 18 days of medication therapy, all mice were sacrificed by cervical dislocation. All animal experiment protocols received authorization from the Animal Care and Use Committee of Tongji University (ID: SYXK 2021-0012, Shanghai, China).

### Statistical analysis

SPSS Statistics version 25.0 was applied to the statistical analysis, with the utilization of an unpaired Student’s *t* test or one-way analysis for multiple comparisons. Data are presented as the mean±standard deviation (SD). *P*<0.05 was set as the significance threshold.

## Results

### DHCE exerts potent antitumor activity in BTZ-R MM cells

**Figure 1:**
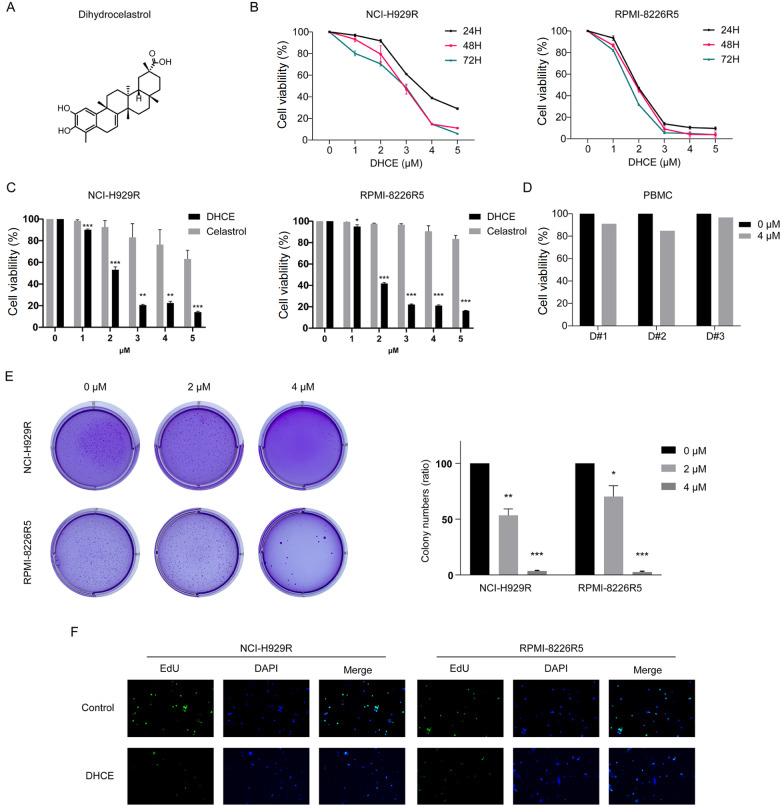
Figure 1 Cytotoxicity of DHCE towards BTZ-R MM cells (A) Chemical structure of DHCE. (B) NCI-H929R and RPMI-8226R5 cells received DHCE (0‒5 μM), and cell viability was evaluated at specified time points. (C) NCI-H929R and RPMI-8226R5 cells received DHCE or celastrol (0‒5 μM) for 48 h before cell viability evaluation. *P<0.05, **P<0.01, ***P<0.001 vs celastrol. (D) PBMCs from three healthy volunteers were exposed to DHCE (0, 4 μM) for 48 h followed by assessment of cell viability. (E) DHCE-treated NCI-H929R and RPMI-8226R5 cell-based colonies in the presence of 0, 2, or 4 μM DHCE and quantification (n=3; *P<0.05, **P<0.01, ***P<0.001). (F) Proliferation micrographs (original magnification, 50×), as evidenced by the EdU assay (DAPI, blue, nuclear staining; EdU, green, proliferating cells).

DHCE, with a molecular weight of 452.6 Da, is an innovative synthesized dihydro-analog substance ( Figure 1A). Previously, we found that DHCE caused dose-dependent cytotoxicity in BTZ-S NCI-H929 and RPMI-8226 cells. To examine the DHCE efficacy in BTZ-R MM cells, RPMI-8226R5 and NCI-H929R cells were incubated with increasing concentrations of DHCE and celastrol. Following 48 h of exposure, cell survival was evaluated via CCK-8 assay. DHCE has greater antitumor activity than celastrol ( Figure 1C), with IC _50_ values of 2.21±0.02 μM and 2.33±0.07 μM in NCI-H929R and RPMI-8226R5 cells, respectively, whereas the IC _50_ values of celastrol were 6.97±1.79 μM and 12.19±2.88 μM. Based on this work, a CCK8 assay, soft agar clonogenic assay, and EdU labeling were performed to focus on the DHCE-mediated anti-proliferative outcomes in BTZ-R MM cells. NCI-H929R and RPMI-8226R5 cells received increasing doses of DHCE for 24, 48, or 72 h. CCK-8 assay showed that DHCE considerably reduced the survivability of BTZ-R MM cells in a concentration-dependent manner ( Figure 1B). A comparable trend was noted in colony formation, demonstrating that DHCE had the ability to inhibit BTZ-R MM cell growth in a concentration-dependent manner ( Figure 1E). In addition, fewer EdU staining were observed after DHCE treatment than in the control group ( Figure 1F). In contrast, even at doses as high as 4 μM, DHCE exhibited a modest cytotoxic effect in normal human PBMCs ( Figure 1D). These findings revealed that DHCE markedly suppressed the proliferation of BTZ-R MM cell lines; however, it was non-cytotoxic to normal cells.

### DHCE induces G _0_/G _1_ phase arrest in BTZ-R MM cells

**Figure 2:**
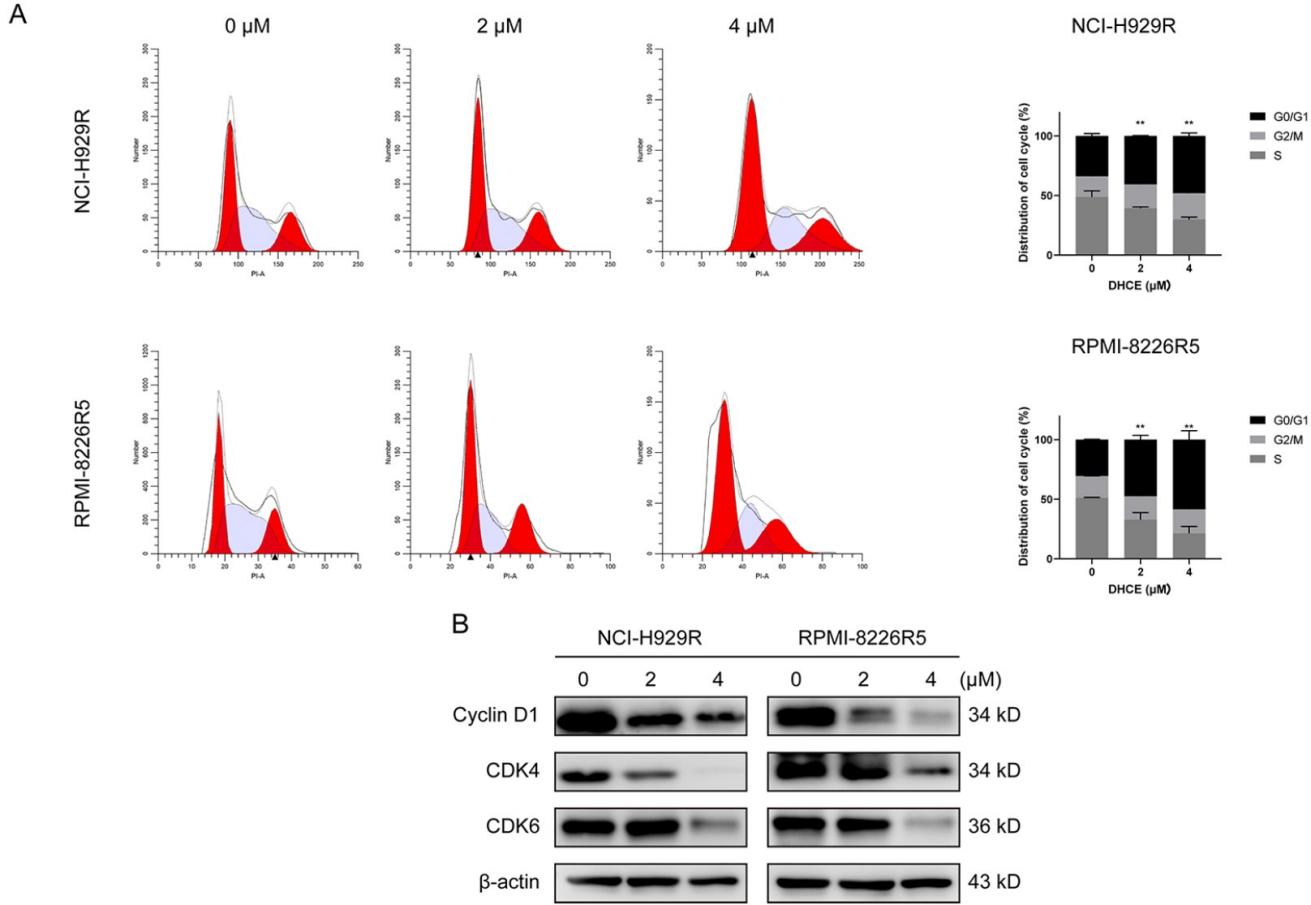
Figure 2 DHCE initiates cell cycle arrest at the G _0_/G _1_ phase in BTZ-R MM cells (A) NCI-H929R and RPMI-8226R5 cells were exposed to DHCE (0, 2, 4 μM) for 48 h prior to PI staining and analysis via flow cytometry. Bar graphs depicting the proportions of G0/G1, S and G2/M phase cells. **P<0.01 relative to the control cells (G0/G 1 stage). (B) After 48 h of treatment with DHCE (0, 2, and 4 μM), proteins isolated from NCI-H929R and RPMI-8226R5 cells were assessed via western blot analysis to detect alterations in the CDK6, CDK4, and cyclin D1 protein contents.

Cell cycle arrest (CCA) plays an essential part in the mechanism of action of antitumor drugs. Cyclin D–CDK4/6 complexes are critical checkpoints from G _0_ /G _1_ to the S phase [17]. Focusing on the impact of DHCE on the cell cycle, we used flow cytometry to examine BTZ-R MM cells after treatment with varying DHCE concentrations (0, 2, or 4 μM). The results showed that increased DHCE level resulted in a considerable rise in the G _0_/G _1_ phase proportion ( Figure 2A). Moreover, DHCE reduced the expression levels of CDK4, CDK6, and cyclin D1 proteins, leading to CCA at the G _1_ checkpoint ( Figure 2B). These findings suggested that DHCE promoted CCA in BTZ-R MM cells at the G _0_ /G _1_ phase.

### DHCE promotes cell apoptosis in BTZ-R MM cells

**Figure 3:**
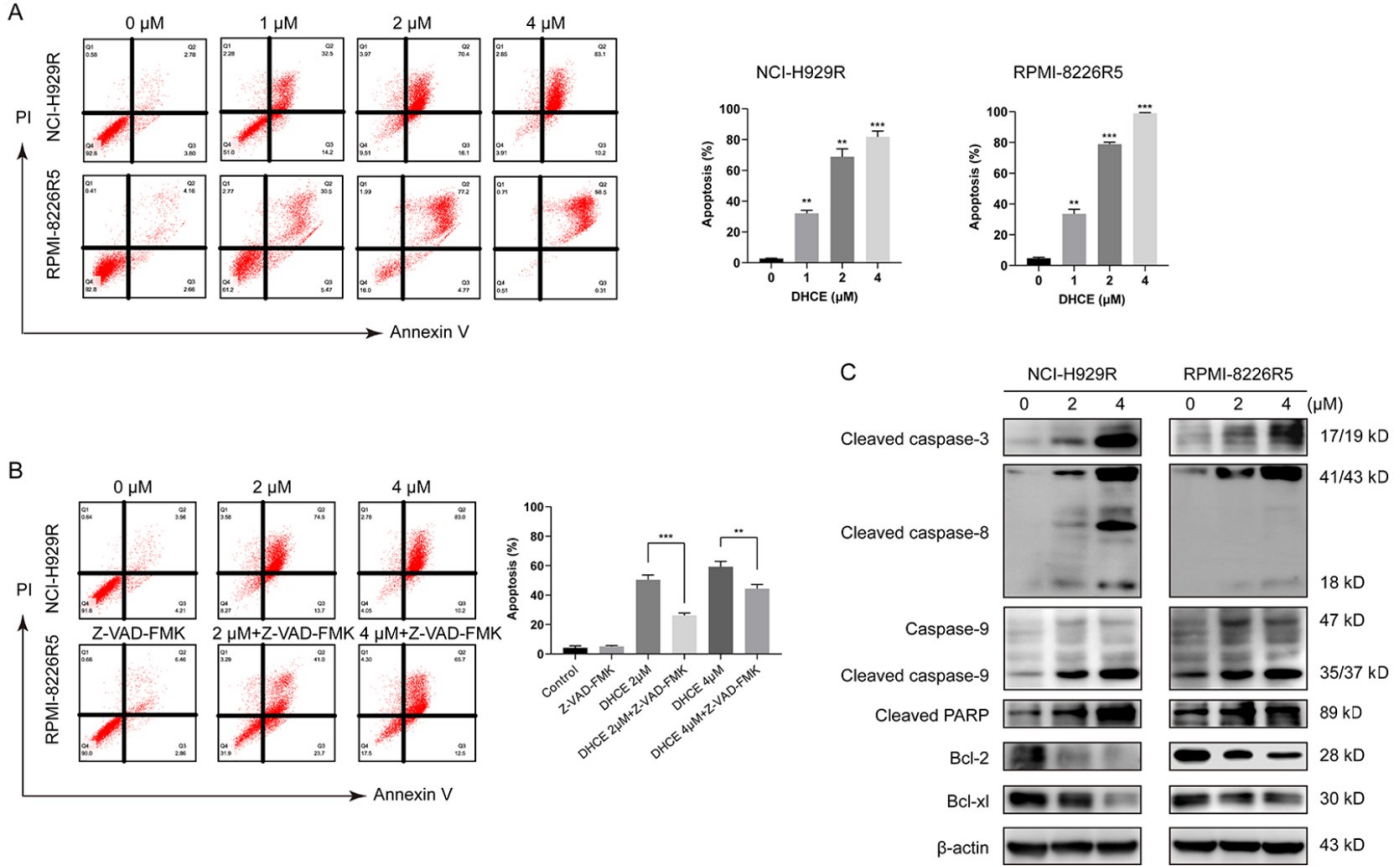
Figure 3 DHCE induces cell apoptosis in BTZ-R MM cells (A) NCI-H929R and RPMI-8226R5 cells were exposed to DHCE (0, 1, 2, and 4 μM) for 48 h, followed by Annexin V/PI staining and FACS. Statistical analysis of apoptosis is presented in the right panel (**P<0.01, *** P<0.001 relative to the control cells). (B) Cells were maintained, with or without the pancaspase inhibitor Z-VAD-FMK, prior to DHCE treatment (0, 2, and 4 μM) for 48 h, with subsequent Annexin V/PI staining and analysis by FACS. Statistical analysis of apoptosis is presented in the right panel (**P<0.01, ***P<0.001). (C) Apoptosis-related protein expression assessment by western blot analysis.

We also investigated the potential relevance between apoptosis and the anti-proliferative activity of DHCE. NCI-H929R and RPMI-8226R5 cells were treated with 0, 1, 2, or 4 μM DHCE for 48 h. Flow cytometry demonstrated that increased DHCE level caused a simultaneous rise in the proportion of late-stage (Annexin-V+/PI+) apoptotic cells ( Figure 3A). To further verify this, Z-VAD-FMK, which functions as a pan-caspase suppressor, was applied for 48 h to NCI-H929R cells with DHCE. As expected, the late-stage apoptotic cell proportion was substantially reduced ( Figure 3B). Furthermore, western blot analysis revealed that the cleaved caspase-3, caspase-8, caspase-9, and PARP protein levels were elevated with increasing DHCE concentrations, whereas the levels of the anti-apoptotic proteins Bcl-2 and Bcl-xl were diminished ( Figure 3C). These results indicated that DHCE activated caspase-dependent apoptosis in a dose-dependent manner.

### Synergism of DHCE and BTZ in BTZ-R MM cells

**Figure 4:**
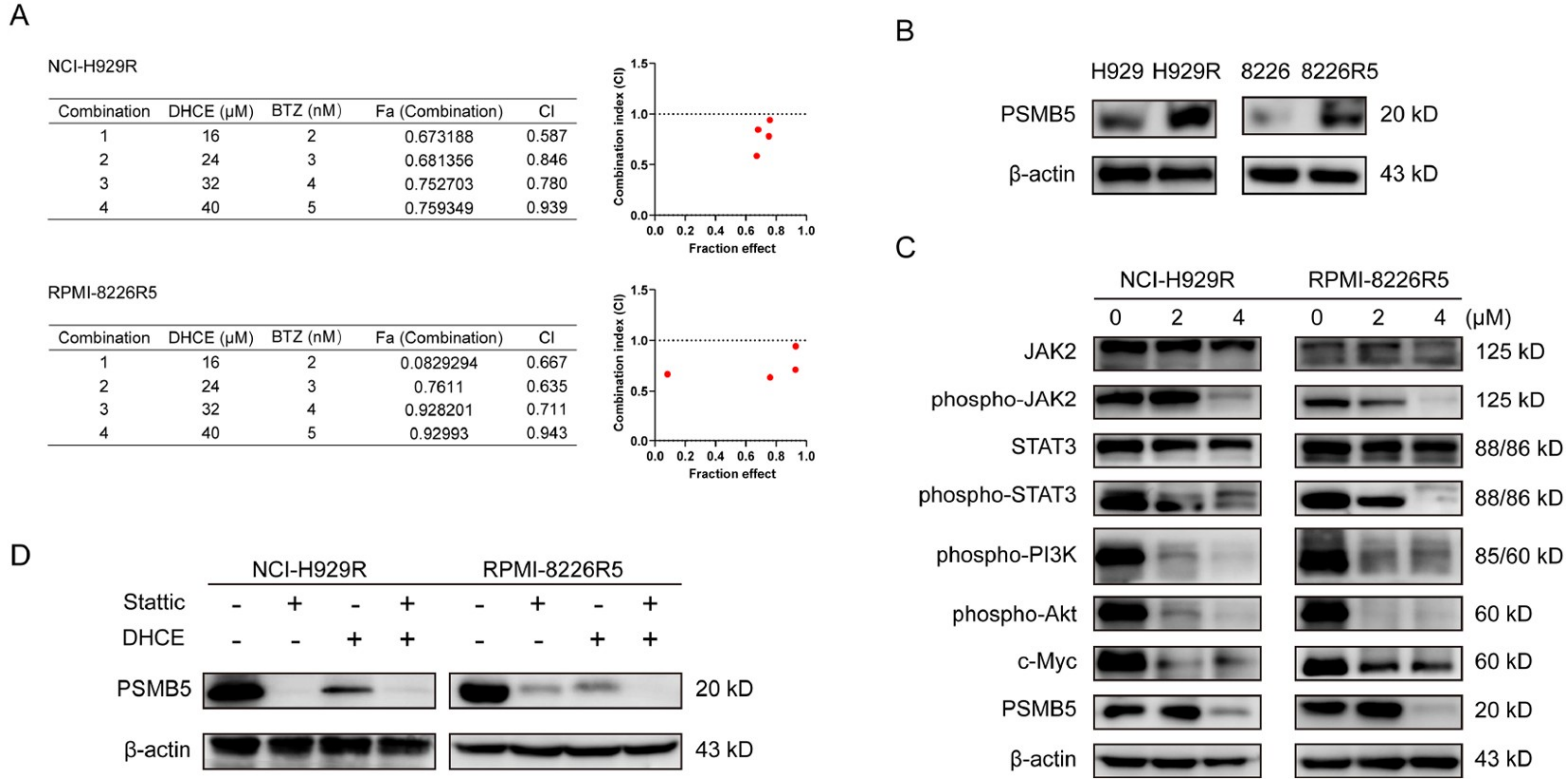
Figure 4 DHCE enhances the sensitivity of BTZ by inhibiting STAT3-dependent PSMB5 regulation (A) NCI-H929R and RPMI-8226R5 cells were exposed to DHCE and BTZ alone or together for 48 h, and then cell survival ability was evaluated. CI values were calculated according to the median-effect principle. CI < 1 indicated DHCE and BTZ synergism, as determined using CalcuSyn software. (B) The PSMB5 protein levels in NCI-H929, RPMI-8226, NCI-H929R and RPMI-8226R5 cells were assessed via western blot analysis. (C) NCI-H929R and RPMI-8226R5 cells received DHCE (0, 2, and 4 μM) for 48 h prior to assessment of JAK2, phospho-JAK2, STAT3, phospho-STAT3, phospho-PI3K, phospho-Akt, c-Myc, and actin protein expression via western blot analysis. (D) PSMB5 expression in BTZ-R cells was assessed via western blot analysis following DHCE (2 μM) treatment with (+) or without (−) STAT3 inhibitor (Stattic).

The latent ability of DHCE to re-sensitize BTZ-R MM cells was explored next. Both DHCE and BTZ were added at a constant ratio to the cell suspension. Interactions between two drugs are estimated using the combination index (CI), and CI < 1 is regarded as the indicator of synergism [ 18, 19]. As shown in Figure 4A, DHCE had clear synergism with BTZ in BTZ-R MM cells, indicating that DHCE could suppress BTZ resistance.

### PSMB5 is overexpressed in BTZ-R MM cells

PSMB5, a subunit of the 26S proteasome, is targeted by the proteasome inhibitor BTZ. According to studies on the molecular mechanisms of BTZ resistance, higher levels of PSMB5 were shown to be associated with resistance to BTZ [ 20, 21]. To find more evidence to support this mechanism in MM, we investigated the PSMB5 protein expression levels in NCI-H929, NCI-H929R, RPMI-8226, and RPMI-8226R5 cells. Western blot analysis revealed that PSMB5 expression in BTZ-R MM cells was significantly higher than that in BTZ-S MM cells ( Figure 4B). These results verified that BTZ resistance in MM is related to the abnormal overexpression of PSMB5.

### DHCE leads to suppression of JAK2/STAT3 and PI3K/Akt

Many studies have identified a series of signaling pathways that promote cell survival and proliferation in MM. To ascertain the possible function of networks that modulate the DHCE-based suppression of MM cell growth, RPMI-8226R5 and NCI-H29R cell proteins were isolated and detected by western blot analysis following 48 h of exposure to varying DHCE concentrations. The results demonstrated that the p-STAT3, p-PI3K, p-Akt, c-Myc, and p-JAK2 protein concentrations in DHCE-treated MM cells were markedly reduced dose-dependently, whereas the total JAK2 and STAT3 levels were unaffected ( Figure 4C). These findings imply that DHCE suppresses BTZ-R MM cell proliferation via inactivation of the JAK2/STAT3 and PI3K/Akt axes.

### DHCE downregulates PSMB5 via STAT3

A study by Vangala *et al*. [22] revealed that PSMB5 is a target of STAT3 that has been tested in other tumor cells. In our study, western blot analysis of NCI-H929R and RPMI-8226R5 cells demonstrated that PSMB5 expression was diminished in DHCE-treated BTZ-R MM cells ( Figure 4C). To examine the relationship between STAT3 and PSMB5 expression, BTZ-R MM cells were exposed to DHCE and the STAT3 inhibitor Stattic alone or in combination. Western blot analysis demonstrated that DHCE treatment decreased PSMB5 protein levels in accordance with Stattic ( Figure 4D). These results indicated that DHCE enhanced BTZ sensitivity in BTZ-R MM cells by inhibiting the JAK2/STAT3 pathway, thus downregulating PSMB5 expression.

### Anti-myeloma effect of DHCE in the BTZ-R MM xenograft model

**Figure 5:**
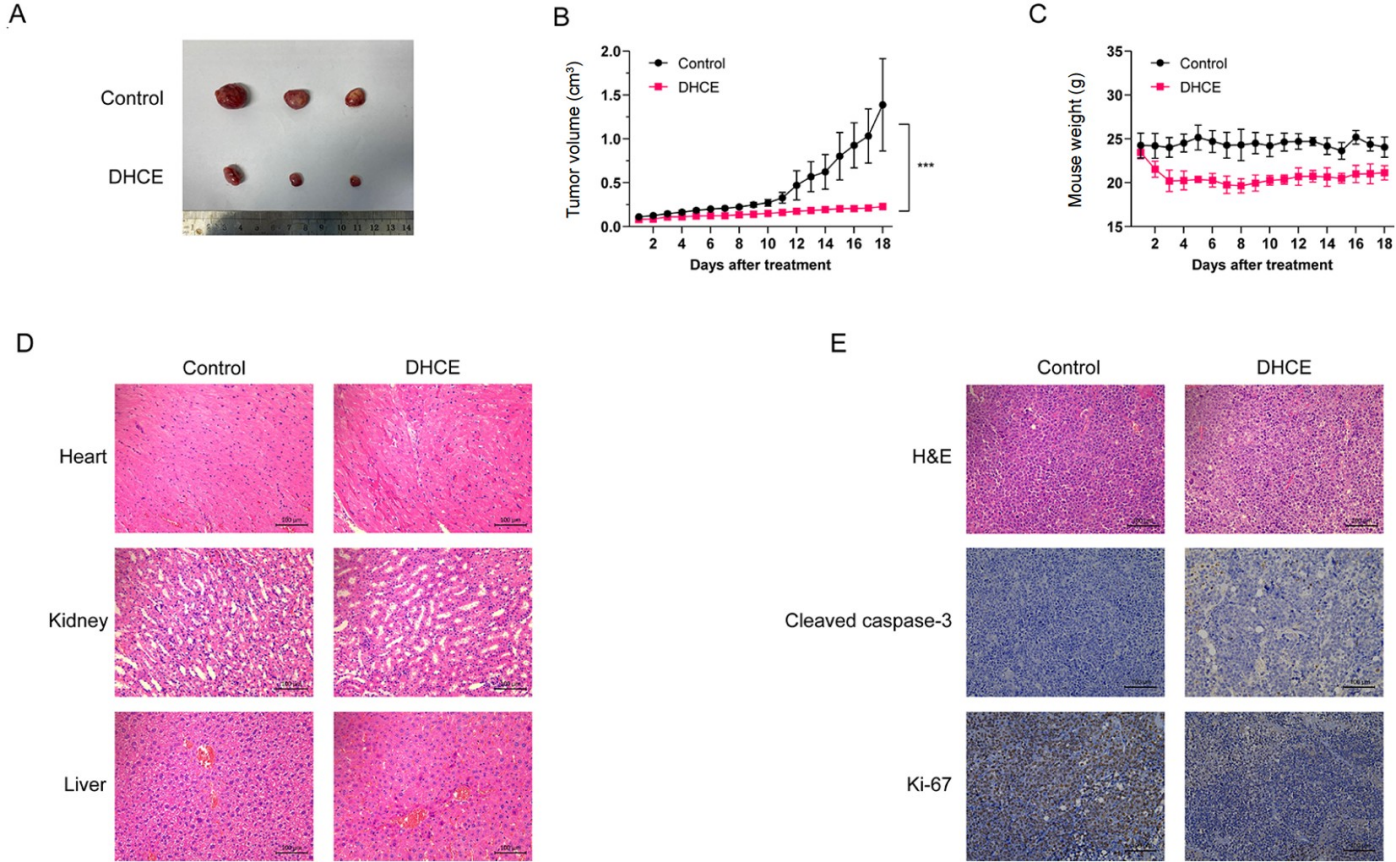
Figure 5 DHCE suppresses BTZ-R cell xenograft tumor development (A) Tumor specimens were photographed with a high-definition digital camera. (B) Nude mice harboring subcutaneous NCI-H929R xenograft tumors were provided with daily intraperitoneal administrations of either DHCE (15 mg/kg) or a vehicle control for 20 days. Mean tumor volume (cm3) and standard deviation are provided ( n=3 mice/group, ***P<0.001 compared to the control group). (C) Body weight was recorded every day for 20 days. Data are presented as the mean±standard deviation. There was no significant difference between the groups (P>0.05). (D) H&E staining of heart, liver and kidney sections excised from DHCE- or vehicle-treated MM-xenograft mice. Scale bar: 100 μm. (E) H&E staining and immunochemical staining of Ki-67 and cleaved caspase-3 of tumor samples from the control and DHCE-exposed xenograft tumor tissues. Scale bar: 100 μm.

To further detect any possible side effects and examine the impact of DHCE *in vivo*, we designed a BTZ-R MM xenograft model in BALB/c nude mice. DHCE (15 mg/kg) were given to the DHCE treatment group by intraperitoneal injection, while the control group received the same volume of saline. After 18 days, the DHCE-treated group showed reduced tumor volume and suppressed tumor growth compared with the control group (0.23±0.01 cm ^3^ vs 1.39±0.74 cm ^3^) ( Figure 5A,B). The DHCE and control groups had no significant variations in mouse weight ( Figure 5C). Meanwhile, there were no significant variations in the cardiac, hepatic, and renal tissue forms between the two groups ( Figure 5D). H&E staining indicated that DHCE substantially decreased the extent of tumor necrosis. Furthermore, immunohistochemistry demonstrated a decline in Ki-67 expression and a rise in cleaved caspase-3 content in the DHCE group ( Figure 5E). The findings in this BTZ-R MM xenograft model showed that DHCE is a potential chemical compound for inhibiting BTZ-R MM tumor growth without exerting lethal toxicity.

## Discussion

Over the past decade, resistance to BTZ has increased along with BTZ-based therapy, which is widely used as an effective therapeutic strategy for MM [23]. DHCE is an innovative dihydro-analog of celastrol synthesized by our team. Our previous study showed that DHCE promoted apoptosis and restrained MM cell proliferation in BTZ-S MM cells via IL-6/STAT3 pathways [14]. In the current study, we probed the potent antitumor activity of DHCE in BTZ-R cells and its ability to counter BTZ resistance. We found that DHCE exerted stronger inhibitory effects than celastrol on BTZ-R MM cells (NCI-H929R and RPMI-8226R5 cells). The cell proliferation assay results revealed that both concentration- and time-dependent inhibitory effects were observed in DHCE-treated MM cells. *In vitro*, cotreatment with DHCE and BTZ demonstrated that DHCE acts synergistically to promote cytotoxicity with BTZ. This experiment validated that DHCE treatment might be a possible therapeutic strategy for the prevention of BTZ resistance in MM.

Apoptosis is a strictly regulated and evolutionarily conserved process that maintains a homeostatic balance between cell survival and death [ 24, 25]. Two different pathways are involved in apoptosis: the extrinsic mechanism which is regulated by death receptors on the cell surface, and the intrinsic mechanism which involves the Bcl-2 family modulating mitochondrial activity [26]. Herein, a series of classic apoptotic traits were examined in DHCE-exposed BTZ-R cells. The flow cytometry results revealed that DHCE caused dose-dependent cell apoptosis in BTZ-R MM cells, which could be rescued from apoptosis by the pan-caspase suppressor Z-VAD-FMK. External and internal apoptotic pathways are both stimulated by DHCE, as evidenced by caspases-8, -3, -9, and PARP protein activation and Bcl-2 and Bcl-xl protein inhibition.

Multiple checkpoints and orderly stimulation of cyclin-dependent kinases (CDKs) govern cell cycle regulation. The cyclin D1-CDK4/CDK6 complex is an essential checkpoint for controlling and regulating cell progression from G _1_ to S phase [ 27, 28]. DHCE treatment downregulated the cyclin D1, CDK4, and CDK6 protein levels, leading to excessive activation of the G _1_ checkpoint, which confirmed that DHCE could cause G _0_/G _1_ phase CCA in BTZ-R MM cells.

The survival and growth of MM cells are influenced by a variety of signaling pathways. These pathways can be divided into three categories: NF-κB pathway, JAK/STAT and MAP-kinase pathways, and PI3K/AKT and MAP-kinase pathways. JAK signaling also activates the PI3K/AKT axis [ 29, 30]. Following treatment with DHCE in BTZ-R cells, the decreased p-JAK2, p-STAT3, p-PI3K, and p-Akt levels indicated that these factors were targeted by DHCE. The experimental results indicated that the observed antitumor effects caused by DHCE are related to the repression of the JAK2/STAT3 and PI3K/Akt axes.

BTZ reversibly inhibits PSMB5, a subunit of the 26S proteasome. According to several studies in different cell lines, higher PSMB5 protein expression is associated with BTZ resistance, and PSMB5 could be regulated by STAT3 [ 20– 22, 31]. Herein, we found that the PSMB5 expression was markedly enhanced in BTZ-R MM cells (RPMI-8226R5 and NCI-H929R cells) compared with that in BTZ-S cells (RPMI-8226 and NCI-H929 cells). In addition, PSMB5 expression is diminished in DHCE-treated BTZ-R MM cells. These results validated that DHCE downregulated PSMB5 overexpression in BTZ-R MM cells. Additionally, PSMB5 level was decreased in BTZ-R cells exposed to the STAT3 inhibitor stattic. This finding supported the theory that STAT3 is an upstream regulator of PSMB5. Combined with the previous study of DHCE inhibiting the JAK2/STAT3 pathway, these experimental results suggested that DHCE counteracted BTZ resistance by inhibiting STAT3-dependent PSMB5 regulation.

Another argument for the possible application of DHCE in MM therapy comes from a nude mouse xenograft model of BTZ-R MM cells (NCI-H929R). Intraperitoneal administration of 15 mg/kg DHCE substantially suppressed cell growth and proliferation without affecting body weight or damaging vital organs. Immunohistochemical analysis of tumor samples confirmed the antitumor activity of DHCE in MM.

In conclusion, DHCE, a novel compound synthesized by our team, induced antitumor activity in BTZ-R MM cells both *in vitro* and *in vivo*. It promotes apoptosis by downregulating activated JAK2/STAT3 and PI3K/AKT pathways. In addition, DHCE sensitizes BTZ-R MM cells to BTZ by inhibiting STAT3-dependent PSMB5 regulation. These findings indicate that DHCE might be a novel therapeutic regimen for BTZ-R MM.

## References

[1] Firth J. Haematology: multiple myeloma.
Clini Med (London, England) 2019, 19: 58–60. https://doi.org/10.7861/clinmedicine.19-1-58.

[2] Twombly R. First proteasome inhibitor approved for multiple myeloma.
J Natl Cancer Inst 2003, 95: 845–845. https://doi.org/10.1093/jnci/95.12.845.

[3] Dimopoulos MA, Richardson PG, Moreau P, Anderson KC (2015). Current treatment landscape for relapsed and/or refractory multiple myeloma. Nat Rev Clin Oncol.

[4] Rajkumar SV (2020). Multiple myeloma: 2020 update on diagnosis, risk‐stratification and management. Am J Hematol.

[5] Richardson PG, Mitsiades C, Hideshima T, Anderson KC (2006). Bortezomib: proteasome inhibition as an effective anticancer therapy. Annu Rev Med.

[6] Kisselev AF. Site-specific proteasome inhibitors. 2021, 12: 54.

[7] Gandolfi S, Laubach JP, Hideshima T, Chauhan D, Anderson KC, Richardson PG (2017). The proteasome and proteasome inhibitors in multiple myeloma. Cancer Metastasis Rev.

[8] Vij R, Wang M, Kaufman JL, Lonial S, Jakubowiak AJ, Stewart AK, Kukreti V (2012). An open-label, single-arm, phase 2 (PX-171-004) study of single-agent carfilzomib in bortezomib-naive patients with relapsed and/or refractory multiple myeloma. Blood.

[9] Yang H, Chen D, Cui QC, Yuan X, Dou QP (2006). Celastrol, a triterpene extracted from the Chinese “thunder of god vine,” is a potent proteasome inhibitor and suppresses human prostate cancer growth in nude mice. Cancer Res.

[10] Yan F, Wu ZH, Li ZH, Liu L. Celastrol inhibits migration and invasion of triple-negative breast cancer cells by suppressing interleukin-6 via downregulating nuclear factor-kappa B (NF-kappa B).
Med Sci Monitor 2020, 26: 9. https://doi.org/10.12659/MSM.922814.

[11] Nagase M, Oto J, Sugiyama S, Yube K, Takaishi Y, Sakato N (2003). Apoptosis induction in HL-60 cells and inhibition of topoisomerase II by triterpene celastrol. Biosci Biotechnol Biochem.

[12] Li HY, Zhang J, Sun LL, Li BH, Gao HL, Xie T, Zhang N,
*et al*. Celastrol induces apoptosis and autophagy via the ROS/JNK signaling pathway in human osteosarcoma cells: an
*in vitro* and
*in vivo* study.
Cell Death Dis 2015, 6: 14. https://doi.org/10.1038/cddis.2014.543.

[13] Kannaiyan R, Hay HS, Rajendran P, Li F, Shanmugam MK, Vali S, Abbasi T (2011). Celastrol inhibits proliferation and induces chemosensitization through down-regulation of NF-κB and STAT3 regulated gene products in multiple myeloma cells. Br J Pharmacol.

[14] Hu L, Wu H, Li B, Song D, Yang G, Chen G, Xie B (2017). Dihydrocelastrol inhibits multiple myeloma cell proliferation and promotes apoptosis through ERK1/2 and IL-6/STAT3 pathways
*in vitro* and
*in vivo*. Acta Biochim Biophys Sin.

[15] Xie Y, Li B, Bu W, Gao L, Zhang Y, Lan X, Hou J,
*et al*. Dihydrocelastrol exerts potent antitumor activity in mantle cell lymphoma cells via dual inhibition of mTORC1 and mTORC2.
Int J Oncol 2018, 53: 823–834. https://doi.org/10.3892/ijo.2018.4438.

[16] Chou TC. Drug combination studies and their synergy quantification using the Chou-Talalay method.
Cancer Res 2010, 70: 440–446. https://doi.org/10.1158/0008-5472.CAN-09-1947.

[17] Otto T, Sicinski P (2017). Cell cycle proteins as promising targets in cancer therapy. Nat Rev Cancer.

[18] Chou TC (2006). Theoretical basis, experimental design, and computerized simulation of synergism and antagonism in drug combination studies. Pharmacol Rev.

[19] Chou TC. The combination index (CI < 1) as the definition of synergism and of synergy claims.
Synergy 2018, 7: 49–50. https://doi.org/10.1016/j.synres.2018.04.001.

[20] Lue S, Chen Z, Yang J, Chen L, Gong S, Zhou H, Guo L (2008). Overexpression of the PSMB5 gene contributes to bortezomib resistance in T-lymphoblastic lymphoma/leukemia cells derived from Jurkat line. Exp Hematol.

[21] Oerlemans R, Franke NE, Assaraf YG, Cloos J, van Zantwijk I, Berkers CR, Scheffer GL (2008). Molecular basis of bortezomib resistance: proteasome subunit β5 (PSMB5) gene mutation and overexpression of PSMB5 protein. Blood.

[22] Vangala JR, Dudem S, Jain N, Kalivendi SV (2014). Regulation of PSMB5 protein and β subunits of mammalian proteasome by constitutively activated signal transducer and activator of transcription 3 (STAT3). J Biol Chem.

[23] Dou Q, Zonder J (2014). Overview of proteasome inhibitor-based Anti-cancer therapies: perspective on bortezomib and second generation proteasome inhibitors versus future generation inhibitors of Ubiquitin-Proteasome system. Curr Cancer Drug Targets.

[24] Shi YG. Mechanisms of caspase activation and inhibition during apoptosis.
Mol Cell 2002, 9: 459-470. https://doi.org/10.1016/s1097-2765(02)00482-3.

[25] Ma R, Yu D, Peng Y, Yi H, Wang Y, Cheng T, Shi B (2021). Resveratrol induces AMPK and mTOR signaling inhibition-mediated autophagy and apoptosis in multiple myeloma cells. Acta Biochim Biophys Sin.

[26] Taylor RC, Cullen SP, Martin SJ (2008). Apoptosis: controlled demolition at the cellular level. Nat Rev Mol Cell Biol.

[27] Icard P, Fournel L, Wu Z, Alifano M, Lincet H (2019). Interconnection between metabolism and cell cycle in cancer. Trends Biochem Sci.

[28] Wang H, Yu D, Zhang H, Ma R, Wu H, Zhai H, Wang H (2021). Quercetin inhibits the proliferation of multiple myeloma cells by upregulating PTPRR expression. Acta Biochim Biophys Sin.

[29] Papadas A, Asimakopoulos F. Mechanisms of Resistance in Multiple Myeloma.
Handb Exp Pharmacol 2018, 249: 251–288. https://doi.org/10.1007/164_2017_10.

[30] Hu K, Li B, Ma R, Yi H, Xu Z, Peng Y, Yu D (2021). Anti-DLBCL efficacy of DCZ0825 and : involvement of the PI3K-AKT-mTOR/JNK pathway. Acta Biochim Biophys Sin.

[31] Wei W, Zou Y, Jiang Q, Zhou Z, Ding H, Yan L, Yang S (2018). PSMB5 is associated with proliferation and drug resistance in triple-negative breast cancer. Int J Biol Markers.

